# Signaling Pathway and Transcriptional Regulation in Osteoblasts during Bone Healing: Direct Involvement of Hydroxyapatite as a Biomaterial

**DOI:** 10.3390/ph14070615

**Published:** 2021-06-26

**Authors:** Junaidi Khotib, Maria Apriliani Gani, Aniek Setiya Budiatin, Maria Lucia Ardhani Dwi Lestari, Erreza Rahadiansyah, Chrismawan Ardianto

**Affiliations:** 1 Department of Pharmacy Practice, Faculty of Pharmacy, Universitas Airlangga, Surabaya 60115, Indonesia; anieksb@yahoo.co.id (A.S.B.); chrismawan-a@ff.unair.ac.id (C.A.); 2 Doctoral Programme of Pharmaceutical Sciences, Faculty of Pharmacy, Universitas Airlangga, Surabaya 60115, Indonesia; maria.apriliani.gani-2019@ff.unair.ac.id; 3 Department of Pharmaceutical Science, Faculty of Pharmacy, Universitas Airlangga, Surabaya 60115, Indonesia; maria-lestari@ff.unair.ac.id; 4 Department of Orthopaedics, Faculty of Medicine, Universitas Airlangga, Surabaya 60132, Indonesia; rahadiansyah@fk.unair.ac.id; 5 Department of Orthopedic and Traumatology, Faculty of Medicine, Airlangga University Teaching Hospital, Surabaya 60115, Indonesia

**Keywords:** neglected diseases, osteoblast transcription factors, osteoblast signaling pathway, Runx2, ERK, p38, Wnt, BMP, osteoblast differentiation

## Abstract

Bone defects and periodontal disease are pathological conditions that may become neglected diseases if not treated properly. Hydroxyapatite (HA), along with tricalcium phosphate and bioglass ceramic, is a biomaterial widely applied to orthopedic and dental uses. The in vivo performance of HA is determined by the interaction between HA particles with bone cells, particularly the bone mineralizing cells osteoblasts. It has been reported that HA-induced osteoblastic differentiation by increasing the expression of osteogenic transcription factors. However, the pathway involved and the events that occur in the cell membrane have not been well understood and remain controversial. Advances in gene editing and the discovery of pharmacologic inhibitors assist researchers to better understand osteoblastic differentiation. This review summarizes the involvement of extracellular signal-regulated kinase (ERK), p38, Wnt, and bone morphogenetic protein 2 (BMP2) in osteoblastic cellular regulation induced by HA. These advances enhance the current understanding of the molecular mechanism of HA as a biomaterial. Moreover, they provide a better strategy for the design of HA to be utilized in bone engineering.

## 1. Introduction

Hydroxyapatite (HA) is a biomaterial used for the production of orthopedic and dental implants. Typically, HA is used as a single material or composite in combination with other materials [1,2,3], a coating agent for bone graft [1,4,5], and a matrix for drug delivery systems targeting bone tissue [6]. HA is widely used because it contains similar physical and chemical components to those of natural HA present in bone tissue [2]. Moreover, studies reported that HA induced a favorable immune response [7], exerted an angiogenic effect on defective bone tissue [8,9], and activated osteoblasts and osteoclasts for bone tissue remodeling by accelerating the differentiation of these cells [10,11].

Osteoblasts are cells that play important roles in bone tissue repair. They increase bone growth at the defect area by synthesizing bone matrix, which is subsequently mineralized [12]. Prior to that, osteoblasts must be differentiated from their precursors. Several transcription factors are vital in osteoblastic differentiation, such as the master transcription factor Runt-related transcription factor 2 (Runx2) and its downstream osterix (Osx) [13,14], activating transcription factor 4 (ATF4) [15], distal-less homeobox 5 (Dlx5) [16], msh homeobox 1 (Msx-1) [17], and Msx-2 [18], and they are responsible for the expression of osteoblast proteins, including alkaline phosphatase (ALP) [19], collagen, and noncollagenous proteins [20,21,22,23,24,25]. These transcription factors are commonly used as markers in studies involving osteoblastic differentiation, including those related to the use of HA for bone regeneration [26,27,28].

Before inducing changes in gene expression in a particular cell, a stimulator, (e.g., HA) activates a signaling pathway. Understanding the role of signaling pathways in cellular regulation helps to determine the physiological basis and identify new therapeutic strategies [7,28]. Several studies reported that the changes in transcription factors induced by HA are mediated by several signaling pathways, for instance, the mitogen-activated protein kinases (MAPK), Wnt, and bone morphogenetic protein (BMP) signaling pathways [26,27,28]. Furthermore, studies also reported that the physical characteristics of HA are also important in determining the cellular mechanism of the osteoblast lineage. Reportedly, differences in HA sizes and particle shapes activated a particular signaling pathway and upregulated specific transcription factors [28]. This also occurred due to the surface topography of HA [26]. This review discusses the signaling pathway and transcription factors associated with osteoblastic differentiation induced by HA. The available evidence enhances the current understanding of the molecular mechanisms of HA as a biomaterial, and provides a better strategy for the design of HA to be utilized in bone tissue regeneration.

## 2. HA

HA (Ca_10_[PO_4_]_6_[OH]_2_) is a derivate of calcium phosphate along with tricalcium phosphate and bioglass ceramic [29]. Calcium phosphate is often used as a biomaterial for the production of bone and dental tissue implants, or as a drug delivery system targeting hard tissue [2,6,9,30]. The organic and inorganic components account for 40% and 60% of the extracellular matrix (ECM) of bone, respectively. The organic component of bone tissue consists of 90% type I collagen (COL1) and 10% noncollagenous protein, whereas the inorganic part consists of bone HA [31]. This renders HA the most suitable biomaterial for bone regeneration [2].

HA possesses biocompatible, biodegradable, osteoconductive, and osteoinductive properties. It is biocompatible because its chemical and physical properties resemble those of natural HA in bone [31]. The biodegradable nature of HA is attributed to its slow degradation when used in vivo, following the growth of new bone tissue [9]. Furthermore, HA also functions as a scaffold to bone tissue, making it osteoconductive, especially with collagen or gelatin. As a composite with collagen or gelatin, HA exhibits similar compressive and tensile strength to that of human bones. These composites also mimic the inorganic-organic component naturally present in bone [11,32]. In addition, HA is an osteoinductive material able to induce osteogenesis, particularly when combined with growth factors and osteogenic cells [33].

The beneficial properties of HA contributed to the in vivo performance compared to other biomaterials. HA extracted from bovine bone caused higher blood vessel formation than tricalcic phosphate and perioglass in vivo [34]. HA also supported new bone growth compared to β-TCP in vivo [35]. Moreover, a study by Lee et al. reported that HA caused greater expression of the ALP and COL1 encoding genes than calcium metaphosphate in a mouse intramuscular defect model [36].

Based on a retrospective radiological study, osteointegration occurred in cranial hydroxyapatite implants to a degree of more than 50% [37]. Furthermore, based on clinical data, HA-coated implants consistently had higher cumulative survival rates at upper molar sites than titanium-coated implants until eight years after placement [38]. Similar results were found in the study by Pieske et al. in which HA-coated pins in external fixators applied for unstable fractures showed a trend towards a superior clinical outcome compared to stainless steel pins [39]. Moreover, another clinical study reported that the application of nanocrystalline HA in human intrabony periodontal defects resulted in improved soft and hard tissue parameters after six months. This was as effective as the use of autogenous bone graft, which is recognized as the gold standard in clinical uses [40]. Therefore, further study and sustainable use of HA have excellent potential in clinical uses.

HA is obtained through synthesis from calcium and phosphate or extraction from natural materials. Synthesis is performed via wet, dry, or high-temperature methods. Each method produces HA with different sizes, morphology, and a calcium-to-phosphorous (Ca/P) ratio [41]. These characteristics are important in determining the compatibility and osteoconductivity of HA when used as a biomaterial. For example, the Ca/P ratio of biomaterials should be similar to that naturally present in bone, which is 1.67 [42]. Compared to other materials, HA possesses great characteristics. For example, HA had greater mechanical strength compared to β-TCP, making it resistant to premature degradation in vivo [35]. Furthermore, the addition of HA to a particular scaffold resulted in greater tensile strength compared to the addition of silica [43].

HA is also obtained from natural sources through extraction from mammals (bovine, camel, and horse), fish and shells, or plants and algae [44]. Mammals, such as bovine, are the most common source of HA owing to its abundance in their bones. Moreover, the chemical content of HA extracted from bovine bone (termed bovine HA) is similar to that of human bones. This renders bovine-HA-based implants suitable for orthopedic and dental uses [2,45]. Different extraction sources also determine the physical properties of HA powder, such as particle size and morphology [2]. Together with the chemical content, the physical properties of HA induce different molecular responses in osteoblasts, which also affect the in vivo performance of HA-based implants [11,28]. For instance, a smaller grain size of HA (nanomaterial) has been associated with better bone matrix synthesis than a larger grain size [46]. Thus, it is important to determine the synthesis method or the natural source and extraction method to obtain HA.

Another important thing that determines the in vivo performance of HA is the grain size. Studies showed that HA fabricated in the nanoscale (<200 nm, nanoHA) enhanced its osteoinductive and osteoconductive properties. The nanoHA was formed using various methods, generally using the hydrothermal treatment method [5,26,27]. HA is a biomaterial that is widely used for bone tissue of different sizes and topographic characteristics. Microscale-sized HA (microHA) is the classical HA used as bone tissue scaffold. The administration of microHA was able to induce new bone growth in the defect area in vivo [9,47]. However, Chandran et al. (2016) showed that the administration of microHA in osteoporotic rats did not provide a higher regeneration efficiency than the sham group [48]. This is also in line with clinical findings [49,50,51]. RCT by Schlagenhauf et al. (2019) showed that daily use of microHA dentifrice on caries progression was not significantly different from 1400 ppm fluoride toothpaste [49]. In addition, HA dentifrice also did not significantly affect plaque formation rate in chronic periodontitis patients compared to fluoridated control. [51]. This then led to comparison studies of nanoHA and microHA. Studies have proven that nanoHA had superior results compared to microHA in vitro [52,53,54]. The superior effect of nanoHA was also proven in in vivo studies [55,56]. One of those is the study conducted by Daugela et al. (2018). The authors reported that nanoHA-based bone scaffold provided higher new bone growth than the microHA in a rabbit calvarial defect model [56]. This proves that despite having the same chemical components, nanoHA has a specific mechanism that makes it superior to microHA. It can be emphasized that nanoHA has the ability to bind to target protein on the cell surface, thereby triggering signaling pathway activities that have an impact on new bone growth.

Nanomaterials have a larger surface area than microscale materials. A large surface area increases the wettability of the material. This was proven by the lower contact angle of nanoHA compared to microHA [46]. nanoHA beneficially contributes to the use of this material in biological systems. Bezerra et al. reported that the wettability of nanoHA increased the adsorption of proteins present in the extracellular matrix of bone tissue [57], for instance, fibronectin [58]. Fibronectin biomaterials interact with bone cells by binding to the integrins present in the cells through the Arg-Gly-Asp (RGD) sequence [58]. This accelerated the differentiation and proliferation of osteoblasts [59,60]. In addition, nanoHA also mimics the size of natural HA found in bone tissue. The formation of HA crystals in bone is still not clearly understood. However, several studies have shown that HA crystal formation begins when osteoblasts deposit crystals to the bone tissue, with a size of approximately 50–200 nm [61,62]. Therefore, nanoHA as biomaterial strongly supports its osteoinductive and osteoconductive properties.

## 3. Osteoblasts, Their Transcription Factors, and Other Marker Proteins

### 3.1. Runx2

The application of HA as a biomaterial for bone regeneration depends on bone mineralizing cells termed osteoblasts. HA induces the activity of osteoblasts, which increases the synthesis of new bone matrix in bone defects [11]. For this purpose, osteoblasts must be differentiated from their precursor cells, termed multipotential stem cells (MSCs) from marrow [12]. Runx2 is a master transcription factor expressed on osteoblast lineage cells and chondrocytes. Osteoblast precursor cells which express *Runx2* are referred to as “preosteoblasts” [63]. Runx2, also termed core binding factor α1 (Cbfa), plays a role in almost all phases of osteoblast differentiation [14]. In the absence of osteoblasts, the skeletal system of *Runx2*^−/−^ mice showed a lack of intramembranous or endochondral ossification [64,65]. Of note, *Runx*^+/−^ mice exhibited skeletal abnormalities [39]. Runx2 also inhibited the differentiation of chondrocytes from mesenchymal cells during embryogenesis [66]. The expression of *Runx2* decreased over time during the process of osteoblast differentiation [67]. However, overexpression of *Runx2* in the late stage of osteoblast differentiation inhibited osteoblast maturation, decreased bone mass, and caused osteopenia and bone fracture [68]. Thus, it is suggested that Runx2 negatively regulates the differentiation of osteocytes from osteoblasts. *Runx2* is one of the most common markers investigated in osteoblastic differentiation studies, particularly for early-stage differentiation. It has been shown that biomaterials, such as HA, induce the differentiation of osteoblasts by upregulating *Runx2* [4,26,27].

### 3.2. Osterix (Osx)

Sp7, also termed Osx, is one of the transcription factors involved in the early stages of osteoblast differentiation. *Osx*^−/−^ mice continued to express *Runx2*, indicating that Osx is a downstream factor of Runx2. *Osx*^−/−^ mice failed to form bone, while their preosteoblasts expressed more chondrocyte markers [69]. This suggests that Osx is essential in preventing chondrocyte differentiation. Overexpression of *Osx* inhibited the late stage of osteoblast differentiation [13]. Osx is also important in bone homeostasis. Inactivation of Osx affects the expression of AT-rich sequence-binding protein 2 (Satb2) gene, which also a transcription factor that regulates the differentiation of osteoblasts. The Satb2 gene was downregulated in *Osx*-null calvaria by activating the promoter region of the Satb2 gene in the GC-rich binding site [70]. Inactivation of Osx in the postnatal period caused defects in osteoblast function, followed by decreased bone formation [71]. Together with Runx2, Osx regulated the unique cartilage matrix-associated protein (Ucma) gene. Overexpression of *Ucma* resulted in accelerated mineralized nodule formation [72]. The expression of *Ucma* was decreased in *Runx2/Osx* double heterozygous embryos, while overexpression of *Runx2* and *Osx* increased the activity of the *Ucma* promoter [72]. In line with *Runx2*, HA also induced the expression of *Osx*, which caused osteoblastic differentiation of osteoblast progenitor cells [73]. Thus, *Osx* is also commonly used as a marker for biomaterials-induced osteoblastic differentiation, including HA.

### 3.3. ATF4

ATF4 is a leucine-zipper transcription factor belonging to the ATF/CREB protein family. The ATF4 gene is expressed during embryonic development and life. In most cells, the ATF4 protein is degraded through ubiquitination. However, this protein was not degraded in osteoblasts [15]. ATF44 plays role in bone homeostasis and osteoblastic differentiation from MSCs. Ablation of the ATF4 gene inhibited osteoblast differentiation and reduced β-catenin levels, whereas *ATF4* overexpression increased β-catenin in vitro [74]. It has been shown that ATF4 and Runx2 regulate the expression of the osteocalcin (OCN) gene in osteoblasts [75]. Of note, ATF4 induced the expression of *OCN* in both osteoblasts and non-osteoblastic cells [76]. However, ATF4 is not a common marker investigated in studies related to osteoblastic differentiation.

### 3.4. Dlx5

Dlx5 is a transcription factor that plays important roles in osteoblast and osteoclast activity. It is widely expressed in developing cartilage and in less mature osteoblasts along with Dlx2 and Dlx6; notably, *Dlx3* is expressed in mature osteoblasts and osteocytes [77,78]. The femur of embryonic *Dlx5*-null mice exhibited a decrease in both total and trabecular bone volume. In osteoblast cell culture, *Dlx5*^−/−^ decreased osteoblast differentiation and proliferation, and downregulated the *Runx2*, *Osx*, *OCN*, and bone sialoprotein 2 (*BSP*) genes. In the femur of *Dlx5*^−/−^ mice, osteoclast activity and the RANKL/osteoprotegerin (RANKL/OPG) ratio were increased [16]. Suppression of 5A signal transducer and activator of transcription (STAT5A) activated Dlx5 and increased osteogenesis in vitro and in vivo [79]. Meanwhile, deletion of STAT5A increased bone mass and bone density, prevented age-related bone loss, and increased bone remodeling in mice [79]. In addition, Dlx5 mediates BMP2-induced *Runx2* expression and osteoblast differentiation by direct binding to the *Runx2* promoter (sequences between −756 and −342 bp) [80]. Along with *Runx2* and *Osx*, biomaterials also induced the expression of *Dlx5*, including HA [81]. These markers determine the stage of osteoblast differentiation induced by the presence of biomaterials.

### 3.5. Msx

Msx1, also termed Hox 7.1, is a transcription factor associated with several tissues during embryonic development, including bone and teeth. Msx1 is a regulator of the *OCN* promoter [82]. This transcription factor modulates the expression of various genes, including genes related to cholesterol synthesis during osteoblast differentiation from human dental pulp stem cells (DPSCs) [17]. *Msx1*-null mutation did not cause endochondral ossification in the mandibular condyle [83]. Alongside Msx1, Msx2 is also involved in the craniofacial skeleton. Msx2 negatively regulates the differentiation of adipocytes by blocking peroxisome proliferator activated receptor gamma (PPARγ) and the CCAAT/enhancer-binding protein (C/EBP) family [16]. In humans, mutations in *Msx2* caused craniosynostosis [84]. The *Msx1* and *Msx2* were upregulated during fracture repair [85]. These genes are also upregulated by several biomaterials, and used as osteoinductive markers in vitro [86,87].

### 3.6. Alkaline Phosphatase

ALP is an ectoenzyme that hydrolyzes monophosphate esters. In humans there are four types of ALP: tissue-nonspecific, intestinal, placental, and germ-cell-specific. Tissue-nonspecific alkaline phosphatase (TNALP) is expressed in bone, particularly by osteoblasts [88]. Physiologically, TNAP hydrolyzes inorganic pyrophosphate, which is an inhibitor of HA formation during mineralization, and provides inorganic phosphate for HA formation [19,88,89]. *Alpl*^−/−^ osteoblasts expressed osteopontin (OPN), OCN, COL1, Runx2, and other osteogenic markers, but do not initiate mineralization in vitro [90]. Meanwhile, *Alpl^−/−^* mice exhibited bone defects [91]. ALP has been widely used in various studies as an early marker of osteoblastic differentiation [28]. The expression of ALP gene was decreased following the upregulation of late markers, such as OCN [19]. This is in line with evidence obtained from several studies. At the same time point, HA downregulated the expression of ALP gene and upregulated that of *OPN*, *OCN*, and *COL1* [5,28]. However, other studies have found that ALP expression was positively correlated with that of osteoblast late markers [1,27]. Periodontal ligament stem cells (PDLCs) cultured on nanosized HA (nanoHA) caused a parallel increase in ALP and OCN genes expression over time [27]. This effect may be attributed to differences in the chemical and physical characteristics of HA.

### 3.7. COL1

Collagen proteins are the main proteins present on the bone matrix. COL1 is the most abundant type of collagen protein, which accounts for 90% of the ECM [23]. In adult bone, collagen is in a dense parallel layer that alternates in orientation, parallel to and orthogonal to the axis of load, with approximately 2 mm intervals. HA crystals are deposited on this collagen matrix [89]. Collagen protein is vital for the structural integrity and mechanical resistance of bone tissue [92]. COL1 and osteoblasts have feedback regulation. Osteoblasts activate the *Col1a1* gene by binding of Runx2 to the promoter region of *COL1* [89,93]. Osteoblasts are also cells that synthesize COL1 [94]. Conversely, COL1 was reported to induce the expression of osteoblastic genes in MSCs [95]. Thus, COL1 is widely used as a bone graft component to induce tissue regeneration in defective bones [92]. Induction of gene expression and protein synthesis of COL1 was found after culturing osteoblasts on several types of materials, including HA. HA (nanosized, HA-coated surface, and HA composite) induces gene expression and collagen protein synthesis in osteoblasts [4,5].

### 3.8. Osteopontin

OPN is an acidic glycophosphoprotein expressed by osteoclasts, osteoblasts, osteocytes, and some inflammatory cells [24,96]. Along with BSP, OPN is a member of the SIBLING (small integrin-binding ligand, N-linked glycoprotein) protein family [97]. The expression of *OPN* depends on Runx2, which activates the promoter of the *OPN* [98]. OPN plays a role in osteoclast activity. However, its physiological function in osteoblasts has not been widely reported [24]. OPN plays a role in forming the sealing zone for the resorption activity of osteoclasts by binding to αvβ3 [71]. It also plays a role in the migration of osteoclasts through αvβ3 and CD44 [99]. HA upregulates the expression and synthesis of OPN with various physical characteristics [1,5,28]. Thus, together with COL1, OPN is a widely used marker of osteoblastic differentiation stimulated by biomaterials in osteoblasts.

### 3.9. Osteocalcin

OCN is a gamma-carboxyglutamate protein expressed by osteoblasts. It is the most abundant noncollagenous protein in bone tissue that binds calcium ions to the bone [20]. Studies reported that absence of OCN in mice results in greater bone mass. This is because the absence of OCN increased bone formation without disturbing the resorption activity [100]. Therefore, OCN level in osteoblasts is a marker of mineral deposition [20]. OCN is also involved in endocrine regulation, such as insulin production and sugar homeostasis [101]. Biomaterials, such as HA, induce osteoblast differentiation which is characterized by an increase in OCN [1,5,26,27]. However, the physiological function of OCN in osteoblasts and bone matrix synthesis remains unclear and warrants further investigation.

### 3.10. Osteonectin (ON)

ON, also termed SPARC (secreted protein acidic and rich in cysteine) and BM40, is a protein that binds to calcium. This protein is expressed in mineralized and non-mineralized tissue [22]. *Sparc*-null mice exhibited low bone formation and a low number of both osteoblasts and osteoclasts, leading to decreased bone remodeling and osteopenia [102]. Although ON was detected in non-mineralized tissues, high expression of ON encoding gene was found in the odontoblasts of developing teeth [103]. ON mutant cells exhibited decreased formation of mineralized nodules and a tendency to differentiate into adipocytes, characterized by an increase in adipogenic markers in vitro [104]. ON is also important in procollagen processing, collagen deposition, and its assembly into the ECM [22]. Similar to other non-collagen proteins, ON is commonly used as a marker in the late stage of osteoblastic differentiation. Moreover, it has been reported that its gene expression is induced by materials such as HA [105].

### 3.11. Osteoprotegerin

OPG is a protein expressed in numerous tissues, especially bone tissue. Previously it was thought that OPG is secreted by B lymphocytes and osteocytes [106,107]. However, deletion of *Tnfrsf11b* (the *OPN* encoding gene) in both cells did not cause significant changes in bone mass. However, deletion of the same gene in osteoblasts increased bone resorption and reduced bone mass in mice [25]. OPG and its ligand RANKL are members of the tumor necrosis factor receptor family and essential in bone resorption activity [108]. The OPG/RANKL/RANK system plays a vital role in the pathological process in bone tissue. The interaction of RANKL and RANK initiates a signaling pathway that activates nuclear factor-κB (NF-κB) and regulates gene expression. In contrast, OPG secretion inhibited the resorption activity of bone osteoclasts by binding to RANKL [108]. Expression of the *Tnfrsf11b* gene increases in cultured osteoblasts after the onset of mineralization [109]. OPG also plays a role in maintaining cartilage integrity. *Tnfrsf11b*^−/−^ mice exhibited progressive loss of cartilage matrix and articular cartilage, indicating severe degenerative joint disease [110]. Expression of the *Tnfrsf11b* is induced by various stimuli, including HA as a biomaterial for bone tissue. Prahasanti et al. reported that use of a scaffold containing HA and stem cells increased the expression of OPG and RANKL in vivo [111].

### 3.12. Bone Sialoprotein 2

BSP is an acidic phosphoprotein that belongs to the SIBLING (Small Integrin-Binding LIgand N-linked Glycoprotein) family. It is expressed on mineralized tissue, including bone tissue, by osteoblasts, osteoclasts, and osteocytes [21]. BSP plays a role in initiating the formation of HA crystals through its polycarboxylate sequence [21,112]. Similar to other noncollagenous proteins, BSP is commonly used as a marker of osteoblast differentiation. Use of biomaterials such as HA increased the expression of the BSP-encoding gene in bone marrow stromal cells (BMSCs) through a specific signaling pathway [26]. Overexpression of the BSP-encoding gene increased osteoblastic differentiation markers, calcium incorporation, and nodule formation in osteoblasts. In contrast, suppression of BSP-encoding gene inhibited the associated markers and nodule formation in vitro [113,114]. In addition, BSP is involved in osteoclast activity. *Ibsp*^−/−^ mice exhibited low bone formation rates that were predicted to occur due to a decrease in resorption activity, marked with lower numbers of osteoclasts [114].

## 4. HA-Induced Signaling Pathways in Osteoblasts

### 4.1. Extracellular Signal-Regulated Kinase (ERK) Signaling Pathway

Protein kinases are proteins which catalyze the transfer of a phosphate group from ATP to one or more side chains of a target protein. Protein phosphorylation controls the enzymatic activity of a protein and its interactions with other proteins or molecules [115]. MAPKs are a family of protein kinases that control a series of cellular events ranging from proliferation to controlled cell death [115]. ERK1 and ERK2 are MAPKs involved in cell differentiation. Signaling pathways involving ERK occur due to the induction of growth factors, cytokines, viruses, small compounds, and others, as well as biomaterials (e.g., HA). Ha et al. reported that the administration of nanoHA increased the expression of OPN and decreased that of ALP in BMSCs and the preosteoblast cell line MC3T3-E1; these effects were mediated by the ERK signaling pathway, but not p38 and JNK [28]. In this event, the highest ERK phosphorylation occurred 1 h following exposure to nanoHA [28]; this was similar to the effect induced by the presence of HA [116]. This event occurred due to the interaction of nanoHA (rod-like shape, 10 nm in width, 100 nm in length) with the fibroblast growth factor receptor (Fgfr) and phosphate transporter (PiT). Blockade of these two membrane proteins caused complete inhibition of changes in gene expression [28].

The activation of the ERK pathway is induced by HA as a single material, as well as HA-coated scaffolds. Jang et al. reported that a nanoHA-coated silk scaffold increased the expression of COL3, fibronectin, OCN, ON, OPG, OPN, ALP, and BMP2 genes on DPSCs. Changes in gene expression occurred due to an increase in ERK activity, particularly after culturing cells with 0.15 g of HA-coated scaffold [105].

The physical characteristics of HA also affected the ERK signaling pathway. Based on the study conducted by Xu et al. micro/nano flake-like HA was the best hierarchical structure in increasing gene expression and osteogenic protein production in MSCs compared with needle-like and rod-like HA. This regulation is mediated by the ERK signaling pathway, but not through p38 or JNK. The cells also exhibited the highest fibronectin adsorption when cultured with micro or nano flake-like HA. This finding suggests that fibronectin may play an essential role in cell and HA interactions, which subsequently induce the ERK signaling pathway [117]. Surface topography also influences cellular regulation of osteoblast due to HA. Xia et al. reported that BMSCs cultured on HA with micro-nano-hybrid surface increased the expression of Runx2, BMP2, BSP, and OCN genes via ERK signaling pathway. Moreover, administration of HA increased cell adhesion, cell viability, and ALP activity. HA with these characteristics provided the best in vivo performance compared to the other types (nanosheet, nanorod, and flat and dense surfaces) [26].

Studies also reported that cell adhesion to materials is influenced by heat energy, which acts as physical stimulation to cells. A three-dimensional-like proliferation pattern was observed in a fibroblast cell line cultured with HA after heat treatment (44 °C for 5 min). This effect was thought to be mediated by p38 activation and involved in cell adhesion to HA [118].

ERK phosphorylation due to HA does not occur exclusively in osteoblasts. Culture of primary human aortic smooth muscle cells with nanoHA increased the expression of Runx2, Osx, and COL1 genes via the ERK signaling pathway. This was confirmed by the administration of an ERK inhibitor prior to cell treatment. This effect may underlie the process of vascular calcification in chronic kidney disease [119].

### 4.2. p38 Signaling Pathway

p38 is a kinase that conveys signals from cytokines and the immune system. This kinase also plays a role in stress response, cell growth and survival, and differentiation of various cell types [115,120]. Moreover, p38 is essential in osteoblast differentiation. Inhibition of p38 signaling on primary calvarial osteoblasts inhibited ALP activity and mineral deposition. In addition, p38 mediates ECM mineralization regulated by ON [121]. Apart from a role in physiological events in osteoblasts, p38 also plays an important role in osteoblast regulation by HA. Use of a nanoHA-coated silk scaffold (0.03, 0.15, and 0.3 nanoHA) increased the expression of osteogenic genes in DPSCs via the p38 signaling pathway. These genes were *COL3*, *fibronectin*, *OCN*, *ON*, *OPG*, *OPN*, *ALP*, and *BMP2* [105]. Similar results were reported in a study conducted by Suto et al. [122]. Use of nanoHA increased the expression of BMP2 via the p38 signaling pathway, but not ERK, in PDLCs. This occurred without changes in the calcium and phosphate concentrations in culture supernatants [122].

In terms of cellular regulation of osteoblasts, p38 exhibited crosstalk with other molecular pathways, including the ERK signaling pathway. These two pathways increased the expression of Runx2, BMP2, BSP, and OCN genes. This was confirmed through pretreatment of cells with p38 and ERK inhibitors [26].

Other nanomaterials, such as nanosized bioactive glass (size: ~20 nm) [123], and gold (Au) nanoparticles (size: 20 nm and 40 nm) [124,125], also activate the ERK and p38 signaling pathways [123,124,125]. This proved that the MAPK pathway is involved in the cellular regulation of osteoblasts induced by a wide variety of biomaterials.

### 4.3. Wnt Signaling Pathway

Wnt is a family of proteins that bind to the seven-pass transmembrane frizzled (FZD) receptors. The Wnt signaling pathway is important in cell determination, proliferation, and differentiation. This signaling is separated into canonical and non-canonical, with the former being the most well studied. This signaling is also termed Wnt/β-catenin signaling due to its dependence on β-catenin. The Wnt/β-catenin signaling is activated by binding to the FZD receptor and coreceptor low-density lipoprotein receptor-related protein 5/6 (LRP5/6). This prevents the phosphorylation and degradation of β-catenin, translating β-catenin into the nucleus and determining the fate of MSC differentiation [114]. Physiologically, Wnt/β-catenin signaling plays a crucial role in promoting osteoblast differentiation and maintaining bone mass [126,127]. Among other Wnt ligands, Wnt10b is involved in osteoblast differentiation by inducing the expression of Runx2, Dlx5, and Osx, and suppresses the adipogenic transcription factors C/EBPα and PPARγ [81].

The involvement of the Wnt signaling pathway in osteogenic differentiation occurs in the presence of HA. Culture of PDLCs on HA with a micro-nano-hybrid surface increased the expression of Runx2, ALP, OCN, cementum attachment protein (CAP), cementum protein (CEMP), LRP5, and β-catenin genes via the Wnt signaling pathway. However, this study did not measure the levels of phosphorylated β-catenin [27]; thus, it is not possible to determine whether the signaling occurs is through the canonical or non-canonical pathways. In addition, Chen et al. found that use of a HA-coated surface (thickness: 100 μm) increased the expression of fibronectin, β1 integrin, vinculin, and paxillin in MSCs, suggesting that the cells adhered to the biomaterial surface [4]. Moreover, the expression of Runx2, Osx, COL1, and OCN genes, as well as ALP activity, were also increased in MSCs, thereby indicating osteogenic differentiation. Pretreatment of cells with Dickkopf-1 (Dkk1) (Wnt signaling inhibitor) confirmed that this event was mediated by the Wnt signaling pathway, particularly the regulation of Wnt10b, β-catenin, Runx2, and Osx [4].

The activation of the Wnt signaling pathway by HA is also influenced by the shape and size of HA particles. Zhou et al. cultured MSCs with strontium-doped HA-coated surface with a nanorod-patterned characteristic. The cells exhibited better adhesion, attachment, spreading, proliferation, and osteogenic differentiation than those shown by cells cultured with a HA-coated surface with a nanogranule-patterned characteristic. This study showed that the proteins regulated by the Wnt/β-catenin signaling pathway were ALP, OPN, COL1, and OCN [5].

The osteoinductive property of HA-based scaffolds may be enhanced by combining HA with other materials, such as Au. Liang et al. reported that HA-Au nanocomposites increased the expression of β-catenin, Runx2, OCN, and OPN genes, as well as ALP activity in cultured MSCs compared with HA as a single material. These effects and the promotion rate of cell mineralization were dependent on the Wnt/β-catenin signaling pathway [1].

### 4.4. BMP Signaling Pathway

BMP and Wnt signaling are two pathways that simultaneously regulate osteoblast differentiation [128]. BMPs are members of the transforming growth factor-β (TGF-β) superfamily and essential for bone homeostasis. More than 30 BMPs are involved in canonical and non-canonical BMP signaling. Unlike non-canonical BMP signaling, canonical BMP signaling depends on Smad [127]. Various studies have shown that the BMP signaling pathway is involved in osteogenic differentiation. Therefore, BMP2 is widely used as a growth factor for tissue engineering [127,129,130].

**Figure 1:**
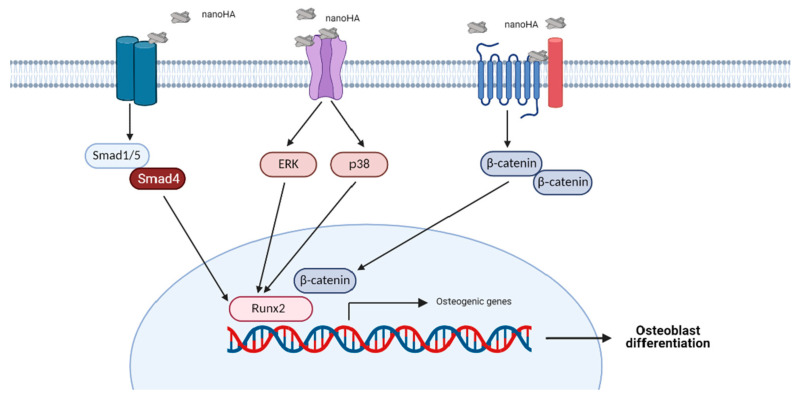
Potential mechanism of the HA-induced signaling pathway. The HA crystals may act as ligands which activate particular signaling receptors and increase the expression of osteogenic transcription factors, indicating osteogenic differentiation.

The BMP signaling pathway also plays a role in osteogenic differentiation induced by HA, particularly in canonical signals (Figure 1). Studies conducted by Tang et al. [131] and Wang et al. [60] found that MSCs cultured with HA (including nanoHA) showed increased expression of osteogenic genes and genes related to the BMP2/Smad signaling pathway, such as *BMPRI*, *BMP2*, *BMP4*, *Smad1*, *Smad4*, and *Smad5* [60,131]. Furthermore, Nahar-Gohad et al. reported a change in gene expression in vascular smooth muscle cells due to the presence of HA. Vascular smooth muscle cells change phenotypes into osteoblast-like cells. Administration of a BMP2 pathway inhibitor blocked the expression of Smad5 and BMP2 proteins in the cells. However, in this study, the expression of other osteogenic genes was not measured after the treatment of cells with a BMP signaling inhibitor [132].

## 5. How Do Bone Cells Produce HA?

Several review articles raised that the physiological HA in the bone tissue is formed by matrix vesicle derived from osteoblast. Specifically, calcium and inorganic phosphate ions are transferred by membrane proteins from the extracellular matrix or catalyzed from lipid-driven phospholipids in matrix vesicles [19,133]. However, there is no clear information regarding the molecular mechanism of this event, such as how calcium and inorganic phosphate ions convert to HA in the vesicle matrix, which is still unclear. It is known that HA produced by bone tissue is rod-like nanoHA that forms clusters [61,62,134]. Some studies that investigate the molecular mechanism of extracellular HA also use HA with these characteristics. The studies found that this rod-like nanoHA activated a particular signaling pathway as previously described (Table 1) [5,28]. Thus, it is indicated that bone HA may also activate a particular signaling pathway in bone cells, particularly osteoblast.

The current understanding of rod-like nanoHA molecular mechanisms might help understand the mineralization process on bone tissue. In addition, this also helps in understanding the pathological mechanisms in the bone tissue or other tissue that were also predicted to be caused by mineralization [132,135]. This hypothesis can be the potential candidate to move forward in further study.

## 6. Other Cellular Events Induced by HA

The activation of signaling pathways due to HA not only affected osteoblast differentiation but also adhesion and proliferation. Zhou et al. (2018) reported that the Wnt/β-catenin pathway was the signaling that mediated the adhesion and proliferation of MSCs induced by HA-coated materials [5]. In addition, Mahato et al. reported that the proliferation of HA-coated glass on osteoblast-like cells was significantly higher than that of non-coated glass [59]. Thus, it is suggested that the increase in cell proliferation rate was due to the presence of HA as intact material.

Furthermore, the particle size of HA also influenced cell proliferation. MSCs cultured on smaller HA exhibited a higher proliferation rate compared to those on larger HA [54]. Not limited to cell proliferation, HA also affected cell adhesion. Xia et al. (2013) reported that HA with nano-structure surface led to better MSCs adhesion and spreading on the biomaterials [26]. This indicated that the proliferation and adhesion of osteoblasts induced by HA were caused by HA as intact material, especially those made in nanoscale.

nanoHA also controversially contributed to cell apoptosis. Remya et al. (2014) demonstrated that nanoHA was not toxic to osteoblasts [136]. However, another study stated that nanoHA inhibited the growth of rat osteoblasts in a dose-dependent manner. nanoHA also significantly induced apoptosis in osteoblasts, with smaller specific surface areas induced lower apoptosis rates [137]. This difference may be due to other topographical cues of HA, such as particle shape. Other than that, this may also be due to the specific signaling pathway that was activated. Therefore, further study of other topographical cues to the activation of signaling pathway is needed.

As reported in the study HA et al. (2015), nanoHA-induced cellular regulation in osteoblast did not occur in cells cultured with nanosized silica [138]. This is also in line with in vivo findings; after 15 days of implantation, nanoHA was superior to nano-bioglass, causing the highest bone formation rate [139]. Thus, we indicated that the cellular response induced by nanoHA was due to the material property of nanoHA.

## 7. Event on the Cell Membrane: Direct Interaction as Intact Ligand or through Ions Release?

As shown in Table 1, HA-induced osteogenic differentiation in the osteoblast cell lineage is mediated by MAPK (particularly ERK and p38 kinases), Wnt, and BMP/β-catenin signaling pathways. However, the mechanism through which HA stimulates the pathway and the events that occur in the cell membrane remain controversial.

As a biomaterial, HA is generally fabricated in nanosizes to resemble the HA found in humans (i.e., approximately 45 nm in length and 25 nm in width) [28,140]. In general, HA that induces cellular changes in the osteoblast linage is nanosized [26,28,105,123]. Materials with a size ≤100 nm are classified as nanomaterials [141]. Osteogenic differentiation occurs at different rates in cells cultured with materials of different particle sizes. HA with a particle size of 40 nm accelerated the expression of osteoblast-like cell osteogenic genes versus HA with a particle size of 0.5–1.0 mm [52]. This is in line with the effects induced by HA with a particle size of ~50 and ~100 nm [53]. In addition, the proliferation rate and ALP activity of MSCs cultured with smaller-sized HA (10–100 nm) were higher than those observed in MSCs cultured with larger-sized HA [54]. This is also consistent with in vivo findings. Freeze-dried bone allografts with smaller particle sizes caused greater new bone formation in Rhesus monkeys with bone defects [55].

The cellular events induced by HA may be mediated by several possible interactions of HA with the cell membrane. For example, HA may release calcium and phosphate from its crystals. Germaini et al. reported that, after 24 h, nanoHA decreased the concentration of calcium in the culture supernatant of a preosteoblast cell line from 1.3 mM to 0.6 mM [10]. It has been reported that high concentrations of calcium affect the resorption activity of osteoclasts [10]. Jung et al. also reported that calcium released by HA is mediated by the calcium/calmodulin-dependent protein kinase via the L-type calcium channel, which subsequently increases the expression of OPN and BSP genes [142]. This theory is supported by the fact that calcium activates MAPK, which plays a role in cell differentiation [143]. However, Ha et al. reported that the calcium channel did not mediate the changes in HA-induced osteogenic gene expression. The study found that the osteoinductive properties of HA were regulated by Fgfr and PiT, which suggested interacting with HA particles [28]. Furthermore, other studies reported that the concentrations of calcium and phosphate in the culture supernatant of cells treated and not treated with nanoHA were not significantly different [122]. This suggests that the cellular regulation that occurs in osteoblasts may be due to the function of intact HA.

Endocytosis is one of the critical events in the interaction of biomaterials with cells. Studies have suggested that HA may be internalized by a particular cell and subsequently regulate its gene expression. Liang et al. conducted a study using transmission electron microscopy and detected HA-Au nanoparticles in endosomal vesicles of MSCs [1]. However, other studies yielded opposite results. Ha et al. found that cellular regulation in osteoblasts is not mediated by endocytosis. This was demonstrated through pretreatment of osteoblasts with several inhibitors of the endocytosis process, such as methyl-β-cyclodextrin, inhibitor of clathrin-mediated endocytosis, and inhibitor of micropinocytosis [28]. This study reported that the ERK signaling pathway was involved in cellular regulation in osteoblasts and mediated by the direct interaction of nanosized HA with receptors on osteoblast cells. The administration of Fgfr and PiT caused a full blockade of p-ERK and changes in gene expression. This result was in line with scanning electron microscopy findings; nanoHA was identified on the surface of osteoblasts even after extensive washing, suggesting a strong interaction between material and cells [28]. This suggests that HA, particularly nanosized HA, may act as a ligand for membrane proteins present in osteoblasts, and activates a particular signaling pathway. However, further study is warranted to confirm this hypothesis.

## 8. Conclusions

Interactions of HA with bone tissue involve a wide variety of cellular events. The physical characteristics of HA influence its osteoinductive properties. NanoHA has been shown to cause cellular changes in osteoblast linage cells. This type of HA induces osteogenic differentiation via several signaling pathways, including ERK, p38, BMP2, and Wnt signaling pathways. It is suggested that these events are mediated by the interaction of nanoHA particles with receptors present on osteoblasts. NanoHA also provides promising results in terms of in vivo performance. These findings offer new insight into the field of biomaterials and a new strategy for the design of HA to be utilized as a biomaterial for bone tissue engineering. However, further research is required to examine this new design strategy.

## Figures and Tables

**Table 1:** Signaling pathways induced by hydroxyapatite (HA).

Signaling Pathway	Upregulated Proteins/Genes	HA Characteristics	Technique	References
ERK	ALP	Micro/nano flake-like HA	qRT-PCR, Western blot	[117]
	BMP-2	Micro-nano-hybrid surface	Pharmacologic inhibitors, Western blot	[26]
	BSP	Micro-nano-hybrid surface	Pharmacologic inhibitors, Western blot	[26]
	OCN	Micro-nano-hybrid surface, micro/nano flake-like HA	Pharmacologic inhibitors, qRT-PCR, Western blot	[26,117]
	OPN	Rod-like shaped (10 nm in width and 100 nm in length)	Pharmacologic inhibitors, Western blot	[28]
	Osx	Nanosized HA (<200 nm)	Pharmacologic inhibitors, Western blot	[119]
	Runx2	Micro-nano-hybrid surface, nanosized HA (<200 nm), micro/nano flake-like HA	Pharmacologic inhibitors, qRT-PCR, Western blot	[26,117,119]
	COL1	Nanosized HA (<200 nm), micro/nano flake-like HA	Pharmacologic inhibitors, qRT-PCR, Western blot	[117,119]
p38	BMP-2	Micro-nano-hybrid surface, nanosized HA (<200 nm)	Pharmacologic inhibitors, Western blot	[26,122]
	BSP	Micro-nano-hybrid surface	Pharmacologic inhibitors, Western blot	[26]
	OCN	Micro-nano-hybrid surface, nanosized HA (<200 nm)	Pharmacologic inhibitors, Western blot	[26,119]
	Runx2	Micro-nano-hybrid surface	Pharmacologic inhibitors, Western blot	[26]
Wnt	ALP	Micro-nano-hybrid surface, nanorod-patterned strontium-doped HA-coated surface (Sr_1_-HA), HA-Au nanocomposites	Pharmacologic inhibitors, Western blot	[1,5,27]
	CAP	Micro-nano-hybrid surface	Pharmacologic inhibitors	[27]
	CEMP	Micro-nano-hybrid surface	Pharmacologic inhibitors	[27]
	LRP5	Micro-nano-hybrid surface	Pharmacologic inhibitors	[27]
	OCN	Micro-nano-hybrid surface, nanorod-patterned strontium-doped HA-coated surface (Sr_1_-HA), HA-Au nanocomposites	Pharmacologic inhibitors, Western blot	[1,5,27]
	OPN	Nanorod-patterned strontium-doped HA-coated surface (Sr_1_-HA), HA-Au nanocomposites	Pharmacologic inhibitors, Western blot	[1,5]
	Osx	HA-coated surface (100 μm in thickness)	Pharmacologic inhibitors, Western blot	[4]
	Runx2	Micro-nano-hybrid surface, HA-coated surface (100 μm in thickness), HA-Au nanocomposites	Pharmacologic inhibitors, Western blot	[1,4,27]
	COL1	Nanorod-patterned strontium-doped HA-coated surface (Sr_1_-HA)	Pharmacologic inhibitors, Western blot	[5]
	Wnt10b	HA-coated surface (100 μm in thickness)	Pharmacologic inhibitors, Western blot	[4]
	β-catenin	Micro-nano-hybrid surface, HA-coated surface (100 μm in thickness), HA-Au nanocomposites	Pharmacologic inhibitors, Western blot	[1,4,27]
BMP	ALP	NanoHA-coated surface	qRT-PCR	[60]
	BMP-2	NanoHA-coated surface, HA	qRT-PCR, pharmacologic inhibitors	[60,131,132]
	BMP-4	NanoHA-coated surface, HA	qRT-PCR	[60,131]
	BMPRI	NanoHA-coated surface	qRT-PCR	[60]
	BSP	NanoHA-coated surface, HA	qRT-PCR	[60,131]
	Dlx5	HA	qRT-PCR	[131]
	OCN	HA	qRT-PCR	[131]
	OPN	NanoHA-coated surface, HA	qRT-PCR	[60,131]
	Osx	NanoHA-coated surface, HA	qRT-PCR	[60,131]
	Runx2	NanoHA-coated surface, HA	qRT-PCR	[60,131]
	Smad1	HA	qRT-PCR	[131]
	Smad4	HA	qRT-PCR	[131]
	Smad5	HA	qRT-PCR, pharmacologic inhibitors	[131,132]
	COL1	HA	qRT-PCR	[131]

Notes: Abbreviations: ALP, alkaline phosphatase; BMP, bone morphogenetic protein; BMPRI, bone morphogenetic protein receptor type I; BSP, bone sialoprotein; CAP, cementum attachment protein; CEMP, cementum protein; COL1, type I collagen; Dlx5, distal-less homeobox 5; ERK, extracellular signal-regulated kinase; HA-Au, hydroxyapatite-gold; LRP5, low-density lipoprotein receptor-related protein 5; OCN, osteocalcin; OPN, osteopontin; Osx, osterix; Runx2, Runt-related transcription factor 2; qRT-PCR, quantitative reverse-transcription polymerase chain reaction; Sr_1_-HA, nanorod-patterned strontium-doped HA-coated surface.

## Data Availability

Data sharing is not applicable to this article.
